# The impact of contextual factors on nursing outcomes and the role of placebo/nocebo effects: a discussion paper

**DOI:** 10.1097/PR9.0000000000000716

**Published:** 2019-06-07

**Authors:** Alvisa Palese, Giacomo Rossettini, Luana Colloca, Marco Testa

**Affiliations:** a Department Biological and Medical Science, University of Udine, Italy, Udine, Italy; b Department of Neuroscience, Rehabilitation, Ophthalmology, Genetics, Maternal and Child Health, University of Genova, Campus of Savona, Italy, Savona, Italy; c Department of Pain Translational Symptom Science, School of Nursing, University of Maryland, Baltimore, MD, USA; d Departments of Anesthesiology and Psychiatry, School of Medicine, Center to Advance Chronic Pain Research, University of Maryland, Baltimore, MD, USA

**Keywords:** Contextual factors, Placebo, Nocebo, Nursing outcomes, Nursing research, Nursing education, Nursing administration

## Abstract

**Introduction:**

Placebo and nocebo effects represent one of the most fascinating topics in the health care field.

**Objectives:**

the aims of this discussion paper were (1) to briefly introduce the placebo and nocebo effects, (2) to elucidate the contextual factors able to trigger placebo and nocebo effects in the nursing field, and (3) to debate the impact of contextual factors on nursing education, practice, organisation, and research.

**Methods:**

a narrative review was conducted based on the available evidence.

**Results:**

Placebo responses (from Latin “I shall please”) are a beneficial outcome(s) triggered by a positive context. The opposite are the nocebo effects (from Latin “I shall harm”), which indicates an undesirable outcome(s) caused by a negative context. Both are complex and distinct psychoneurobiological phenomena in which behavioural and neurophysiological changes arise subsequent to an interaction between the patient and the health care context.

**Conclusion:**

Placebo and nocebo concepts have been recently introduced in the nursing discipline, generating a wide debate on ethical issues; however, the impact on nursing education, clinical practice, nursing administration, and research regarding contextual factors triggering nocebo and placebo effects has not been debated to date.

## 1. Introduction

Placebo and nocebo effects represent one of the most fascinating topics in the health care field. Historically, placebo has been conceptualized as an inert treatment given in clinical randomized trials to compare the efficacy of an active treatment arm vs the placebo arm.^32,95^ The modern neurobiological theories differentiate between placebo and nocebo responses (eg, clinical trials) and placebo and nocebo effects as the neurobiological phenomena independent from biases, regression to the mean, natural history, and co-interventions.^32,95^ Overall in this discussion paper, we define placebo and nocebo effects as the result of patient's interaction with the health care context. Namely, the term “placebo effects” (from Latin placēbō, “I shall please”) have been adopted to describe a beneficial outcome(s) produced by positive contexts,^9^ whereas nocebo effects (from Latin nocēbō, “I shall harm”) have been defined as undesirable outcome(s) produced by negative contexts surrounding the patient and the treatment delivery.^15^

In medical literature, the concept of placebo effects has been debated since 1941,^69^ specifically in the field of medication administration, and was introduced as a Medical Subject Heading (MeSH) in 1990. On the other hand, the concept of nocebo effects was introduced by Kennedy in 1961 and recognised as a MeSH only in 2014. In the nursing discipline, while placebo concept was introduced in 1966,^122^ nocebo was formally introduced in the literature only 8 years ago.^103^ Moreover, both concepts have given rise to a wide debate mainly regarding ethical issues,^5,56^ despite them being proposed as promising clinical tools useful in modulating nursing outcomes.^85,112^

In recent years, the investigation of placebo and nocebo effects have been included the evaluation of the context supporting the patient in achieving (or not) the desired health outcomes.^33^ As a consequence, the misleading interpretation of placebo as inert treatment given to comfort or please the patient has been overcome; conversely, the modern conceptualization of the placebo and nocebo effects as the psychosocial context that accompanies any health care intervention, be it active or sham, has been embraced.^52,100,121^

The context is composed by the “whole atmosphere around the therapy”^6^ created by the health care team, technologies and settings. Recently, specific contextual factors have been proposed in the literature as potential triggers for placebo and nocebo effects.^100,114^ As suggested by many authors,^7,16,36,41,86^ these factors are embodied at different levels: (1) the provider and the patient features (eg, expectations), (2) the patient–provider relationship (eg, empathy), (3) the intervention (eg, the colour and shape of a medication), and (4) the health care setting (eg, the home or hospital room layout). These factors constitute the therapeutic ritual and healing symbols surrounding the encounter able to trigger placebo and nocebo effects, impacting the patient's physiology and psychology and, ultimately, influencing the expected clinical outcome.^10,11^

Considering that a boost of placebo and prevention of nocebo would be valuable in nursing practice, this discussion paper is aimed at (1) briefly introducing the placebo and nocebo effects, (2) elucidating the contextual factors capable of triggering placebo and nocebo effects in the nursing field, and (3) debating implications at the nursing educational, practical, organisational, and research levels.

## 2. Placebo and nocebo effects

Placebo and nocebo effects have been used as a model to investigate the human body systems, analysing their interaction with different systems, mechanisms, diseases, and therapeutic interventions.^8^ Specifically, placebo and nocebo effects have been studied in psychiatric conditions, cardiovascular, respiratory, gastrointestinal, motor, immune, and endocrine systems.^47,48,101^ However, pain represents the most investigated symptom of placebo and nocebo effects.^15,34^ Determinants explaining placebo and nocebo effects have been identified at the individual, psychological, and neurobiological levels.

### 2.1. Individual and psychological determinants

At the individual level, early stages of research aimed at evaluating the role of some genetic variants established as relevant in placebo and nocebo effects, but the available findings are not conclusive.^61^ Preliminary evidence focusing on goal-seeking behaviours, self-efficacy/esteem, the locus of control, optimism, fun seeking, sensation seeking, neuroticism, trust, beliefs, and body consciousness suggested that these can all act as trait predictors of placebo effects.^66^ On the other hand, anxiety, panic disorder, or pessimism can exacerbate nocebo effects.^112^ However, more evidence is available regarding the role of psychological determinants such as expectations and learning.^31,38^

An expectation represents a conscious deliberately reportable element through which the patient expects a beneficial or harmful outcome based on the evaluation of contextual factors such as verbal instructions (eg, communication associated with interventions provided by the nurse) or past experiences (eg, previous interaction with a nurse).^38^ Expectations are able to modify experiences. Anxiety can be further influenced by emotional and cognitive factors, such as self-efficacy, self-reinforcing feedback, memory, attention, and motivation.^8,48,97^

Learning encompasses associative, social, and reinforced expectation mechanisms.^31^ Associative learning emerges when a conditioned neutral stimulus such as a contextual factor (eg, the colour of a medication) is associated with an unconditioned stimulus (eg, the active molecules contained in the medication), and it is responsible for altering the symptom even when the active principle is not administered.^37^ Social learning occurs, for example, when a patient on a specific treatment learns to change symptoms by appreciating the effects reported by other patients.^31^ Moreover, in accordance with Colloca,^31^ expectations and learning represent an interactive phenomenon, given that learning can increase expectations or develop new ones.

### 2.2. Neurobiological determinants

From a neurobiological point of view, placebo and nocebo effects have been documented to be accounted for by a specific neurochemistry and neural network.^101^ Placebo and nocebo effects interact with the brain modulatory systems at a neurochemical level, through the release of specific neurotransmitters.^101^ For instance, considering pain outcome as a model, the endogenous opioids, dopamine, cannabinoids, oxytocin, and vasopressin are involved in the reduction of pain (eg, placebo analgesia) whenever the patient interacts with positive contextual factors.^9,25^ Conversely, cholecystokinin, dopamine, opioid deactivation, and cyclooxygenase-prostaglandins' activation are involved in the amplification of pain (eg, nocebo hyperalgesia) during health care patient encounter surrounded by negative contextual factors.^15,25^

Furthermore, recent advances in neuroimaging techniques, such as functional magnetic resonance imaging and positron emission tomography, suggest an involvement of specific neural correlates during placebo and nocebo effects of pain.^102^ The positive and negative use of contextual factors are capable of activating or inactivating the 4 key brain regions commonly associated with the descending pain processing pathway: the dorsolateral prefrontal cortex, the rostral anterior cingulate cortex, the periaqueductal gray matter, and the spinal dorsal horn.^25,26^ Nevertheless, other classical pain-related areas have been reported to change in their activity during placebo or nocebo effects such as the thalamus, insula, somatosensory cortex, and midcingulate regions.^52,121^

### 2.3. The trigger role of contextual factors

**Figure 1.**
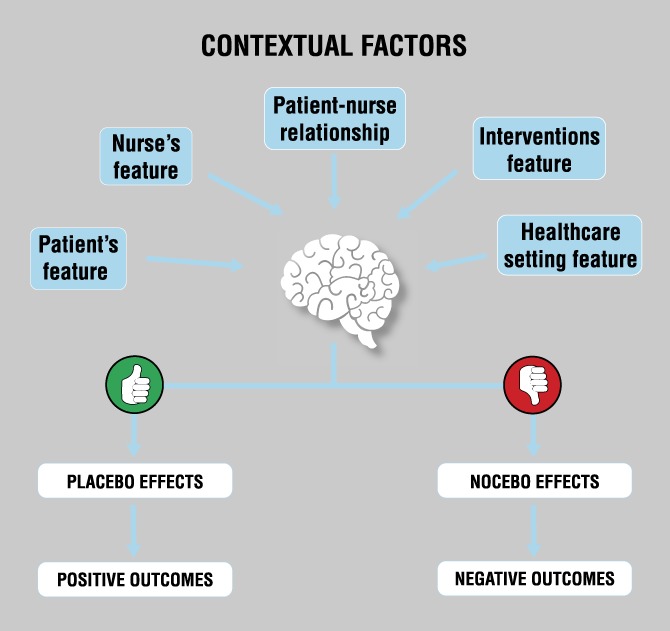
Contextual factors as triggers of placebo and nocebo effects. Adapted from Blasini et al. (2017).

Contextual factors have been documented as triggering placebo and nocebo effects.^100,114^ Specifically, all clinical interventions have been defined as composed by 2 inseparable elements: (1) the first is the intervention itself (eg, the medication and the treatments) mainly based on biological elements, whereas the (2) second is based on the context.^9^ Context is not an empty dimension, but it represents a powerful healing space enriched by emotional, cognitive, affective, social, and relational factors. It is, furthermore, capable of interacting with the patient's clinical condition.^121^ The contextual factors convey a hidden meaning, detected and actively analysed by the patient, which is essential for the perception of care and the interpretation of the therapeutic intervention.^37^ When these factors are analysed from the patient's perspective, they are translated into a complex cascade of psychoneuroimmunoendocrine events capable of generating placebo/nocebo effects and eliciting expectations, memories, and emotions that, in turn, can influence the patient's health-related outcome as presented in Figure 1.^37,121^

Many studies^25,26,71,84,121^ have defined the context and related factors within placebo research, and recently, some have attempted to disentangle each factor,^41^ so that this knowledge can be translated in many areas of health science. For example, Testa and Rossettini^114^ have attempted to identify each factor applying the related knowledge to the field of physiotherapy and musculoskeletal pain.^100^ These contextual factors, capable of influencing clinical outcomes, have been identified as professional reputation, appearance, beliefs, and behaviours of health care providers; expectations, preferences, previous experience, clinical conditions, sex, and age of the patient; verbal and nonverbal elements of communication characterising the patient–health care provider relationship; the environment, architecture, and internal design of the health care setting; and the specific aspects of treatment such as a clear diagnosis, an overt therapy, observational learning and patient-centred approach, a global process of care and the therapeutic touch.

## 3. Subjective nursing outcomes can be modulated by contextual factors

Nursing outcomes are defined as those changes subjectively or objectively reported by patients or by their caregivers and/or family members as a result of the nursing care received.^58^ Safety and efficacy outcomes have been categorised, and specific indicators have been established. Among safety outcomes, falls, pressure sores, hospital acquired infections (eg, pneumonia and surgical site infections), and medication errors leading to death have been described.^58^ Among efficacy outcomes, independence in activities of daily living as well as patient or family self-management competence, coping, comfort, and satisfaction with nursing care have been established and included in several national and international quality indicators and research projects.^58^

With the final purpose of preventing safety outcomes and of achieving efficacy outcomes, clinical nurses develop a plan of care after identifying actual or at-risk problems. On the basis of patient and caregiver preferences, values, and resources, clinical nurses decide the nursing interventions required. These interventions should be based on the available evidence and can consist of simple interventions (patient mobilisation), bundle interventions (such as the prevention of care-associated infections), or complex interventions (such as those performed to improve functional independence in nursing homes). All these interventions can be performed by the same nurse (as in the case of a family nurse or primary nurse) or by a team (as in the case of hospital-based nursing care).

Although evidence emerging from research tries to predict the likelihood of preventing a certain safety outcome or achieving the effective outcome after a specific intervention, it is always necessary to take into account the “uncertainty principle”.^33^ Paraphrasing Colloca and Benedetti,^33^ it is challenging to measure with a significant degree of accuracy the contribution of each intervention (eg, the effect of an educational session) on outcomes as the intervention itself is influenced by contextual factors. Effects determined by interventions have been conceptualized as the sum of the contextual factors effect plus the active intervention effect plus the interaction of the contextual factors and active intervention effects.^119^ Thus, the contextual factors surrounding the patient have the power to interact with the intervention, modulating its effect and outcomes,^25,26,121^ mainly influencing the experience and perceptions of illness symptoms, instead of changing the pathophysiology of disease.^83^

Positive contextual factors can increase the effectiveness of the intervention, whereas negative contextual factors can decrease it.^47^ As a consequence, contextual factors embody an unavoidable component of nursing care responsible of influencing the overall patients' outcomes,^83^ such as (1) the positive patient's experience with care (eg, satisfaction, involvement, empowerment, adherence, and compliance to treatments, motivation, willingness, hope, safety, and the perception of the quality of care); and (2) the symptoms experienced (eg, pain, discomfort, anxiety, nausea, stress, fatigue, social, psychological, physical, and spiritual well-being).

**Figure 2.**
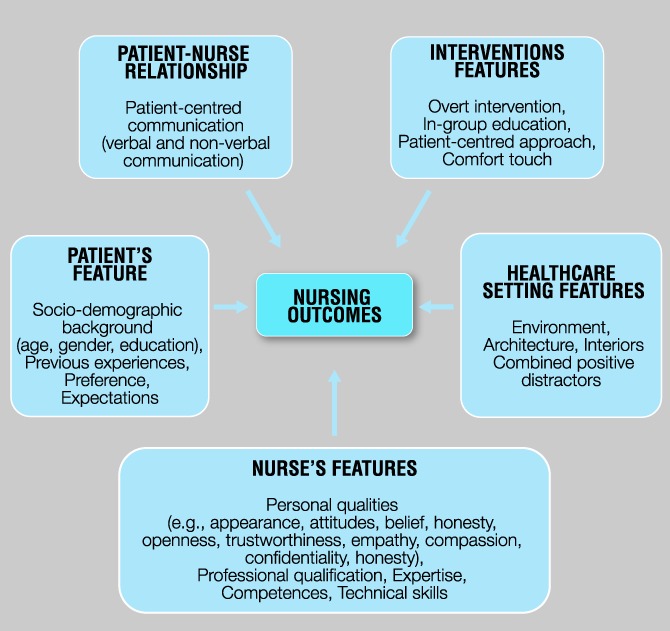
Contextual factors as modulators of the nursing outcomes. Adapted from Testa and Rossettini (2016).

To our knowledge, no attempt has been made to develop an evidence-based clinical toolkit^40^ aimed at summarising contextual factors relevant in placebo and nocebo effects in the nursing disciplines. Thus, the theoretical frameworks about the role of context proposed by different authors^7,16,25,26,36,41,71,84,86,100,114,121^ for the different areas of health science have been considered and translated for the nursing field. A thorough analysis of the contextual factors led to categorize them into: (1) nurse's and patient's features, (2) the patient–nurse relationship, (3) interventional features, and (4) the characteristics of the health care setting, as reported in Figure 2.

Table 1 reports a summary of contextual factors triggering placebo and nocebo effects that clinical nurses should consider in their daily care.

**Table 1:**
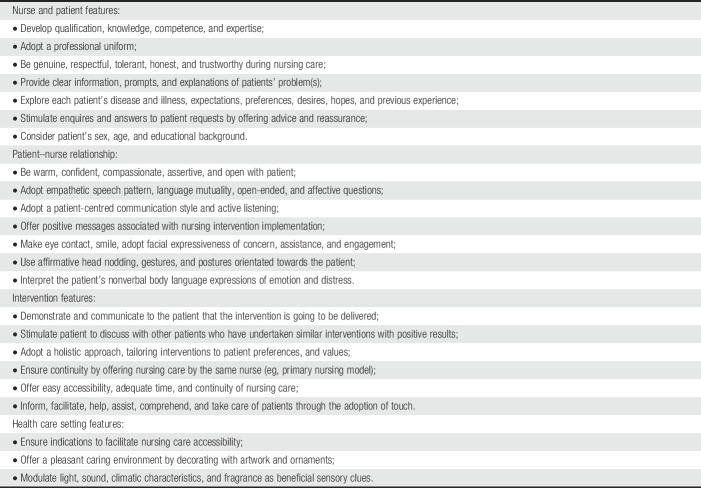
Contextual factors increasing placebo effects and minimizing nocebo effects: a summary for clinical practice (adapted from Testa and Rossettini 2016).

### 3.1. Nurse's features

Nurses embody a specific “effect” because they convey information to the patient through appearance and behaviour that communicate the essence of nursing care. At a first glance, a nurse's uniform is capable of influencing the perception of nursing professionalism and competence by patients.^64,73,96,115^

Professional qualifications, expertise, competences, and technical skills of nurses can influence patient satisfaction, treatment adherence, and compliance with care.^14,43,68,82,125,130^ Moreover, personal qualities such as leadership, attitudes, and beliefs are components known to influence patient satisfaction and the overall experience of nursing care.^120,128,129^ Other personal qualities include honesty, candour, trustworthiness, empathy, compassion, confidentiality, and commitment to providing the best care. Authenticity, assertiveness, humility, and the ability to provide holistic care have also been associated with patient satisfaction and perception of quality with nursing care.^99,117^ Moreover, awareness of unvoiced needs by encouraging patients to share their concerns has also been documented as influencing outcomes. When caring for patients and caregivers, demonstrating compassion, tolerance, and respect, accepting patient preferences and decisions, and providing information regarding illness and health processes, all influence nursing outcomes.^14,43,68,99,117,125^

On the other hand, failure to anticipate or recognise patient needs, depersonalising the patient by referring to him or her using the medical diagnosis or bed number, neglecting care responsibilities, in which patients feel abandoned, vulnerable, ashamed, ignored, or insecure can negatively affect nursing outcomes by increasing the occurrence of safety issues.^43,55,62,99^

### 3.2. Patient's features

The patient's previous experiences, preferences, and expectations are significant elements influencing both placebo and nocebo effects.

The expectations regarding an intervention can determine the patient's involvement, satisfaction, and experience as well as the outcomes regarding pain control.^91,126,131^ Positive expectations, desires, and hopes may increase nursing outcomes,^91,126^ while, as reported recently by Woo,^131^ negative expectations of discomfort during wound dressing changes have been associated with an increased occurrence of pain (also known as nocebo hyperalgesia), wound exudate, and occurrence of necrotic tissues.

Moreover, previous experience of care and preferences can also influence outcomes.^106^ According to a recent systematic review, the likelihood of nocebo effects is increased when previous negative knowledge or expectations exist. In addition, pre-existing psychological traits (eg, anxiety) may exacerbate the nocebo effect.^112^

Furthermore, the sociodemographic background of patients such as age and sex has also been documented as contributing to nursing outcome. Elderly patients have been reported to be more satisfied with nursing care; moreover, men report a higher level of satisfaction with nursing care compared with women, similarly to patients with lower education levels.^70^ On the other hand, the nocebo effects have been reported more often among women.^112^

### 3.3. The patient–nurse relationship

A patient-centred communication based on verbal and nonverbal strategies positively influences the clinical encounter between the nurse and the patient, improving satisfaction, empowerment, adherence to care treatments, trust, pain catastrophizing, stress, fear, anxiety, and symptom resolution.^29,53,78,98^ Differently, as documented recently by Doyle et al.,^44^ poor patient outcomes occur when the nurse's behaviour is callous and lacks empathy.

Verbal communication is emphasised by open-ended and affective questions, the ability to clarify, summarise, and negotiate as well to listen actively; moreover, nonverbal communication is also considered a key factor influencing nursing outcomes such as those techniques aimed at increasing the understanding of the patient's concerns, eg, communicating with empathy, paraphrasing, and following-up cues.^80,90,103^ For example, verbal communication used by nurses during medication administration has been documented to influence the patient's satisfaction and symptoms: pain is influenced positively by verbal suggestion of amelioration and negatively by verbal sentences of aggravation.^2,88,90,109,118^

In addition, the nurse's ability to interpret nonverbal body language expressions of emotion and/or distress may affect patient satisfaction.^80,90^ Tailoring nonverbal communication to patients' sensory deficits is crucial: deaf patients report an increased sense of vulnerability, a risk of delayed recognition of their symptoms and needs, and in receiving appropriate interventions.^108^

### 3.4. Intervention features

Several elements of nursing interventions can affect patient outcome(s). Showing or telling a patient that an intervention is being undertaken can stimulate placebo effects.^13^ Moreover, presenting information about side effects of treatment in form of probability instead of a mere list, as well as balancing positive and negative information can all reduce the nocebo effects.^124^

Creating a therapeutic context in which patients can share their experiences with other patients or can watch videos where other patients report their positive experience with the same intervention may increase the likelihood of positive effects of the treatment.^102^ Similarly, reducing exposure to patients experiencing side effects of the medication can reduce nocebo effects.^72,104,124^ In addition, in the field of educational interventions, offering in-group sessions by nurses instead of individual interventions can trigger some effects^105^: according to the literature available, patients who receive education sessions in a group can increase motivation, willingness, compliance, and hope, and also positive interactions with other participants.^89^ Educational interventions have also been documented to improve pain, anxiety, stress, satisfaction with nursing care, and to reduce side effects of medications.^45,50,54,60,134^

This patient-centred approach is also able to modulate nursing care effects. Tailoring nursing interventions by considering the patient's preferences and needs, empowering them in self-care management and ensuring continuity of care, as well as offering visits by the same nurse, can all positively influence outcomes such as patient's satisfaction, compliance, anxiety, depression, quality of life, and experience with care.^4,14,23,39,43,51,68,94,99,116,125^ On the other hand, higher workloads, long waiting times, the use of medical and sophisticated language, or the use of nurse-centred approaches with a lack of patient understanding and proximity can hamper patient satisfaction.^4,23,43,55,68,99^ In addition, the above-mentioned factors can positively influence the agreement between patients and nurses regarding the care plan, thus increasing its quality.^93^

The comfort touch adopted by nurses has also been recognised as a contextual factor. Touch represents the basis of social interaction conveying information about the emotional and mental state of individuals involved in the relationship.^75^ In nursing care, touching has been documented as a useful intervention that alleviates pain, anxiety, depression, sleep disturbances, nausea, and fatigue, thus increasing quality of life.^3,18,19,30,46,57,92,113,132^ While touching patients, nurses communicate empathy, compassion, affection, concern, and security, thus facilitating the achievement of the expected outcomes.^75^

### 3.5. Health care setting features

Sensory cues, structural aspects, decorations, and ornaments are the most important elements of the healing environment that should be considered when planning and designing the care settings.^22,28,125^

In general, clear indication of health care settings can improve health care accessibility.^59^ Specifically, environments with natural lighting (eg, full-spectrum lighting), low noise levels (eg, adoption of sound-absorbing ceilings or earplugs/earmuffs), and relaxing and soft sounds (eg, music, bird songs, rain showers, and ocean waves) have been documented as greatly appreciated by patients and capable to improve outcomes of anxiety, pain, delirium, satisfaction, and emotional well-being.^1,20,21,42,76,79,107,110,133^ Also the adoption of pleasant aromas and an adequate temperature and microclimate (eg, filters, airflow control, and ventilation systems) all create a positive therapeutic setting.^42^

Moreover, environments that integrate windows and skylights, with comfortable and private settings (eg, single-bed or private patient rooms) have also been documented as positive elements by patients.^27^ Nature artwork, such as flowers or green vegetation in nursing homes, can have a calming effect, thus improving pain, delirium, sleep, and satisfaction.^20,27,42,79^ The presence of healing gardens close to wards (eg, plants and water) and social spaces (eg, lounge, day rooms, and waiting rooms) have been documented as increasing connections between patients and their caregivers, thus reducing stress and pain and promoting well-being. Colour frames based on calming tones also mitigate patient involvement in nursing care; however, the meaning of colour is culturally based and can differ between patients.^42^

Finally, combining positive distractors has been documented as modulating pain, stress, anxiety, and safety, promoting social, psychological, physical, and spiritual well-being.^74^ However, uncomfortable, frightening, oppressive, claustrophobic, and dirty rooms have been associated with patient dissatisfaction and negative outcomes.^24,67^

## 4. Implications for the nursing discipline

Although the placebo effects have a longer history in the nursing literature,^85,122^ the recent introduction of the nocebo effects^103,112^ suggests that these concepts require complete consideration at different levels of the nursing discipline, eg, from education to clinical practice, nursing administration, and research. Because of the relationship with the patient's clinical outcome, it is necessary to identify future directions for inquiry and application starting with a critical evaluation of current nursing practices.

### 4.1. Nursing education

Although the concept of caring as learnt during nursing education embodies several of the above-mentioned factors,^123^ these are not always clearly introduced in nursing programs.^81^ Specifically, if contextual factors are not taught during theoretical lessons and experienced during clinical rotation, there is a risk to consider them as irrelevant by students and faculty members, thus reducing their aware clinical application.^35^ Thus, the nursing programme should consider the contextual factors as core components of the curricula.

Students at different levels of education (from bachelor's and master's nursing degrees) with different degrees of nursing competence should be coached to analyse and consider the relevance of contextual factors in influencing nursing outcomes.^16^ They should be trained to progressively increase their awareness of their own attitudes and traits. In addition, they should learn to develop complex competences in assessing patient and caregiver needs and preferences, also taking into consideration their sociocultural context (eg, culture and ethnicity).

Students should have the opportunity to reflect on how their personal qualities evolve based on clinical experiences.^49^ On the other hand, supervisors at both the faculty and clinical levels should consider student aptitudes and qualities (such as honesty) as specific traits on which students need to receive feedback with the aim of promoting their personal and professional growth. Students should also have the opportunity to reflect with experienced nurses on unexpected negative patient outcomes, by identifying the relevant contribution of some contextual factors in addition to other well-known mechanisms such as nurse-to-patient ratio or physiopathology mechanisms.

Moreover, the clinical context in which students undertake their rotations should promote their learning processes: contextual elements triggering placebo and nocebo effects can also have a role in student learning outcomes, preventing or facilitating their achievements, an area that has not attracted the attention of researchers to date.

### 4.2. Nursing clinical practice

The manner in which clinical nurses consider the contextual factors triggering nocebo/placebo effects in their daily practice has not been extensively documented.^85^ In those clinical settings in which nurses work in groups as in hospitals, shift after shift, the variability of the adoption regarded contextual factors may offset the positive effects obtained or reinforce negative ones. Patients switching from one nursing team to another at the end of shifts, or transiting from one context to another (the medical unit to rehabilitation unit), can experience uncertainty or confusion even if nursing interventions are similar. These effects can be different due to different contextual factors.

Moreover, experienced clinical nurses may identify other factors not clearly included in the available frameworks^87^ by their clinical wisdom and expertise, as well as their close relationship with patients, and may develop an in-depth knowledge of other factors that may modulate the relationship between nursing care and patient outcomes. Therefore, it is ideal to ensure continuity in care by providing the same nurse (eg, primary nursing model of care delivery). When this is not possible, it is advisable for care plans to contain documentation regarding relevant contextual factors and their clinical effects, aiming at ensuring consistency across shifts and contexts to increase the likelihood of a positive nursing outcome.

Clinical nurses should also be supported in developing and maintaining their competences by carrying on educational strategies not only concerning interventions but also the context in which they are implemented. Moreover, with regard to the ethical implications of the contextual factors, clinical nurses have been documented to consider placebo effects as real, with therapeutic benefits, and acceptable within the ethical borders in daily practice.^63^ Therefore, the elicitation of placebo and the avoidance of nocebo effects by contextual factors have been considered ethical.^100,114^ In fact, the conscious use of contextual factors symbolises a useful chance to improve evidence-based nursing care without threatening the principle of nonmaleficence, the patient's autonomy, and informed consent. Therefore, this approach is markedly different from that replacing the required treatment with a potentially ineffective treatment.^17,47,48^

### 4.3. Nursing care management

The clinical settings have received increased attention in recent years as mediators of the quality of nursing care.^111,127^ Examples may be detected in recent studies where the organisational support perceived by clinical nurses may modulate patient outcomes by increasing or decreasing their occurrence.^65,77^ Differently, in light of placebo/nocebo effects, contextual factors may directly influence patients and promote (or hamper) the achievement of expected outcomes. Therefore, the role of the environment should be considered not only as affecting the performance of the nursing workforce, but also as relating to patient outcomes with direct effects.

Nurse leaders should be prepared to continually assess, design, and promote interventions to improve the quality of the environment while cooperating with other leaders both at the hospital, at the community and at the residential levels. There is also a need to develop and validate instruments capable of measuring the quality of factors implied in placebo/nocebo effects, aiming at monitoring the amelioration of the contextual factors over time.

Any form of nursing care standardisation, addressing patients' needs without considering preferences, expectations, and unique needs, should be detected early and avoided. Specifically, some models of nursing care delivery (eg, functional models) should be immediately replaced with person-centred models of care delivery where evidence-based approaches, capable of identifying the best interventions within those documented in the literature, are implemented in an appropriate environment, capable of maximising the effects of the intervention delivered.

### 4.4. Nursing research

Although placebo and nocebo effects are well documented, there has been minimal research in the nursing field.^85,112^

Designing and implementing a trial for placebo and nocebo investigation represents a challenge, and several confounding factors should be controlled.^119^ The history of disease, the influence of uncontrolled biases, unidentified co-interventions, and adverse side effects can all modulate nursing care outcomes.^12^ Research on placebo and nocebo should adopt placebo ethically as an enhancing strategy associated with the best evidence-based available interventions to prevent nocebo and improve nursing outcomes.^17,48^ Moreover, research on placebo and nocebo effects should be based on contextual factors effect.^100,114^ Limiting the influence of the contextual factors around the intervention can help to identify the specific effect of the intervention itself. On the other hand, boosting the context around an active intervention can disclose the role of contextual factors in modulating clinical outcomes.^13^

Different lines of research can be designed and promoted in specialist (eg, critical care nursing, oncology care, mental health, or chronic care) or in general areas, at national and international levels, also considering the cultural differences that may affect placebo and nocebo effects. First, there is a need to explore the knowledge and expertise on placebo and nocebo effects both among undergraduates, registered and advanced nurses, aiming at assessing their awareness in the field and promoting improvements to increase the latter. There is also a need to discover the effect of single and/or combined contextual factors affecting nursing care outcomes, possibly through incremental study designs to weigh the effect of each component. Finally, exploring patient perceptions regarding the contextual elements capable of positively or negatively influencing expected nursing outcomes, as well as researching psychological and genetic traits of placebo and nocebo responders given the documented variability across patients, is recommended.^114^

## 5. Conclusions

To our best knowledge, this is one of the first discussion paper deliberately linking the conceptualization of contextual factors as triggers of placebo and nocebo effects to the nursing discipline, practice, managements, education, and research. The ultimate goal is to raise awareness about the potential effects of contextual factors, placebo and nocebo effects on clinical outcomes managed by nurses.

Contextual factors can trigger positively or negatively the achievement of nursing outcomes. Besides appropriate evidence-based interventions, nurse educators, clinicians, leaders, and researchers should pay further specific attention to contextual factors to develop and to unveil their mechanisms of action by considering their implementation in daily practice. The theoretical framework developed for nurses can be easily generalized to pain medicine in general.

## Disclosures

The authors declared no potential conflicts of interest with respect to the research, authorship, and/or publication of this article.
